# Light Deprivation-Induced Inhibition of Chloroplast Biogenesis Does Not Arrest Embryo Morphogenesis But Strongly Reduces the Accumulation of Storage Reserves during Embryo Maturation in Arabidopsis

**DOI:** 10.3389/fpls.2017.01287

**Published:** 2017-07-20

**Authors:** Huichao Liu, Xiaoxia Wang, Kaixuan Ren, Kai Li, Mengmeng Wei, Wenjie Wang, Xianyong Sheng

**Affiliations:** ^1^College of Life Sciences, Capital Normal University Beijing, China; ^2^Department of Chemistry, Capital Normal University Beijing, China

**Keywords:** *Arabidopsis thaliana*, chloroplast, embryogenesis, storage reserves, oil body, protein storage body

## Abstract

The chloroplast is one of the most important organelles found exclusively in plant and algal cells. Previous reports indicated that the chloroplast is involved in plant embryogenesis, but the role of the organelle during embryo morphogenesis and maturation is still a controversial question demanding further research. In the present study, siliques of Arabidopsis at the early globular stage were enwrapped using tinfoil, and light deprivation-induced inhibition of the chloroplast biogenesis were validated by stereomicroscope, laser scanning confocal microscope and transmission electron microscope. Besides, the effects of inhibited chloroplast differentiation on embryogenesis, especially on the reserve deposition were analyzed using periodic acid-Schiff reaction, Nile red labeling, and Coomassie brilliant blue staining. Our results indicated that tinfoil enwrapping strongly inhibited the formation of chloroplasts, which did not arrest embryo morphogenesis, but markedly influenced embryo maturation, mainly through reducing the accumulation of storage reserves, especially starch grains and oil. Our data provide a new insight into the roles of the chloroplast during embryogenesis.

## Introduction

Plastids are among the most important organelles found exclusively in plant and algal cells. Their functions are crucial to photosynthesis, as well as fatty acid and amino acid synthesis. Moreover, they are considered to be involved in numerous aspects of plant growth and development (Inaba and Ito-Inaba, 2010; Puthur et al., 2013). Therefore, the biological functions of plastids have received considerable attention.

Embryogenesis is a complex and intriguing event, which can be divided into the stages of embryo morphogenesis and embryo maturation. It has long been recognized that the embryos of some angiosperm taxa contain chloroplasts, which are referred to as chloroembryos (Puthur et al., 2013), in which the chloroplast shows specific patterns beginning from the later globular stage and ending with the maturation phase (Tejos et al., 2010). Furthermore, a considerable number of studies revealed that mutations in the chloroplast-related genes usually resulted in the abnormal embryogenesis (Hsu et al., 2010; Inaba and Ito-Inaba, 2010; Bryant et al., 2011; Feng et al., 2014; Kremnev and Strand, 2014; Chen et al., 2015). All these findings outline the important roles of the chloroplast during embryogenesis.

However, the data available at present appear insufficient to provide complete knowledge of the functions of the chloroplast during embryogenesis. For example, mutations in different chloroplast-related genes usually exert similar effects on chloroplast development, but embryo developmental arrest might occur at varied stages. In fact, chloroplast-related mutations might result in an arrest of the embryo at the globular stage, or at the globular-to-heart-shaped transition stage (Trosch and Jarvis, 2011; Yin et al., 2012; Lu et al., 2014; Chen et al., 2015). Chloroplast-related mutants with normal embryo development until the heart stage or torpedo stage have also been established (Liang et al., 2010; Wang et al., 2010; Feng et al., 2014). In other mutants, developmental arrest did not occur until after seed germination, leading to the development of albino seedlings (Hsu et al., 2010; Bryant et al., 2011; Kremnev and Strand, 2014). Given the important roles of plastids during embryo development, and the reversible interconversion of chloroplasts and other types of plastids (Lopez-Juez and Pyke, 2005; Hsu et al., 2010; Inaba and Ito-Inaba, 2010), the relations between defective chloroplasts and embryo developmental arrest are still a controversial problem demanding further research. Furthermore, although many chloroplast-related mutants have been identified with embryonic lethal phenotype (Bryant et al., 2011), little attention has been paid to the possible roles of this organelle in reserve deposition, including that of storage proteins, lipids and starch, all of which are closely linked to both seed formation and germination.

Chloroplast differentiation and chlorophyll biosynthesis are light dependent processes (reviewed in Solymosi and Schoefs, 2010). In chlorenchymatic tissues of plants or organs growing under continuous dark conditions proplastids are usually converted into etioplasts instead of chloroplasts. However, literature data about plastid differentiation in light-deprived reproductive organs and embryos is very scarce.

To extend our knowledge of the roles of the chloroplast in embryogenesis, in the present study, siliques of Arabidopsis at the early globular stage were enwrapped using tinfoil. Light deprivation-induced inhibition of chloroplast biogenesis was validated by a series microstructural and ultrastructural observations. Besides, comparative studies between the control and dark-forced embryos were also carried out to evaluate their embryogenesis, with emphasis on the reserve deposition during embryo maturation.

## Materials and Methods

### Plant Materials and Growth Conditions

Seeds of *Arabidopsis thaliana* (Col-0) were surface-sterilized and cultivated on 1/2 MS medium supplemented with 1% sucrose and 0.8% agar. The resulting seedlings were cultivated in cube pots containing soil mixture of vermiculite and peat (1:1). All samples were cultivated in an artificial climate box with a 16-h-light/8-h-dark cycle, and a day/night temperature of 23°C/21°C. Light deprivation was performed from day 3 after artificial pollination (DAP), by enwrapping the siliques of the third to seventh flowers on the main flowering stems with tinfoil at the early globular stage.

### Observation of Embryo Development

Previous reports indicated that chloroplasts were not observed until late-globular embryos were formed (Mansfield and Briarty, 1991). To determine the developmental stages of the embryo, ovules taken from the middle of the siliques at the early developmental stage (1–5 days after pollination, DAP) were cleared according to a previous report (Lu et al., 2014). Briefly, dissected embryos were immersed in HCG solution (80 g chloral hydrate, 10 ml glycerol, 30 ml H_2_O), and differential interference contrast (DIC) images were obtained immediately using a Zeiss Imager M2 microscope equipped with a Zeiss HRC CCD. From 6 to 14 DAP, ovules/embryos obtained from the siliques were dissected and observed directly under a Zeiss stem SV 11 stereomicroscope equipped with another Zeiss HRC CCD.

### Detection of Chlorophyll in the Chloroplast

Chlorophyll fluorescence is a specific marker for plastids differentiated into chloroplasts (Tejos et al., 2010; Feng et al., 2014; Lu et al., 2014). To clarify the effects of light deprivation on chlorophyll biosynthesis, both control and treated embryos at the developmental stage from 6 to 14 DAP were analyzed using a Zeiss 5 live laser scanning confocal microscope (Zeiss 5 LSCM). Chlorophyll was excited at 488 nm and the emissions were recorded in the range of 575–700 nm. Laser power and channel settings were maintained identical for all samples to ensure comparability of results.

### Nile Red Staining for Oil Body

Nile red staining was performed according to the procedure described in previous reports (Greenspan et al., 1985; Siloto et al., 2006; He and Wu, 2009), with some modifications. Briefly, dissected embryos were infiltrated with 5 μg/mL Nile red (Invitrogen, final concentration) in the dark for 30 min. Images were acquired using the Zeiss 5 LSCM. Under the 488 nm excitation light, the Nile red emissions (within 495–555 nm) were recorded. Similarly, all images were obtained with same laser power and channel settings to ensure comparability of the results.

### Histochemical Staining for Polysaccharide and Protein

Histochemical staining of semithin sections was performed according to the protocol detailed in our earlier report (Sheng et al., 2011). Briefly, dissected embryos at the developmental stage from 6 to 14 DAP were fixed for 1 h in ice-cold 0.1 M phosphate buffer (pH 7.2) containing 2.5% paraformaldehyde and 0.2% glutaraldehyde. Then, the embryos were embedded in LR White resin (Sigma) and polymerized by UV at 4°C for 24 h. To determine the distribution of polysaccharides and proteins during embryo development, sections (1 μm) were obtained with a Leica EM UC6 ultramicrotome that were stained using periodic acid-Schiff (PAS) reaction (Sigma) and Coomassie brilliant blue R250 (Sigma) staining, respectively. All samples were observed under the Zeiss Imager M2 microscope.

### Transmission Electron Microscopic (TEM) Observation

For Transmission Electron Microscopic (TEM) observation, both control and dark-forced embryos were fixed with 2.5% paraformaldehyde and 0.5% glutaraldehyde. A part of 10-DAP embryos was subsequently double-fixed with 1% osmic acid. All embryos were finally embedded in LR White resin. Ultrathin (70 nm) sections were obtained on the Leica EM UC6 ultramicrotome using a diamond knife (Diatome), which were then stained with uranyl acetate and lead citrate (Sheng et al., 2006). All sections were observed under a Hitachi-7650 transmission electron microscope.

### Statistics

All experiments were performed at least in triplicate. One-way ANOVA was used to compare the quantitative difference between the control and treated samples. Values of *P* < 0.05 were considered statistically significant.

## Results

### Light Deprivation Did Not Inhibit Embryo Morphogenesis, but Affected Embryo Maturation

**FIGURE 1:**
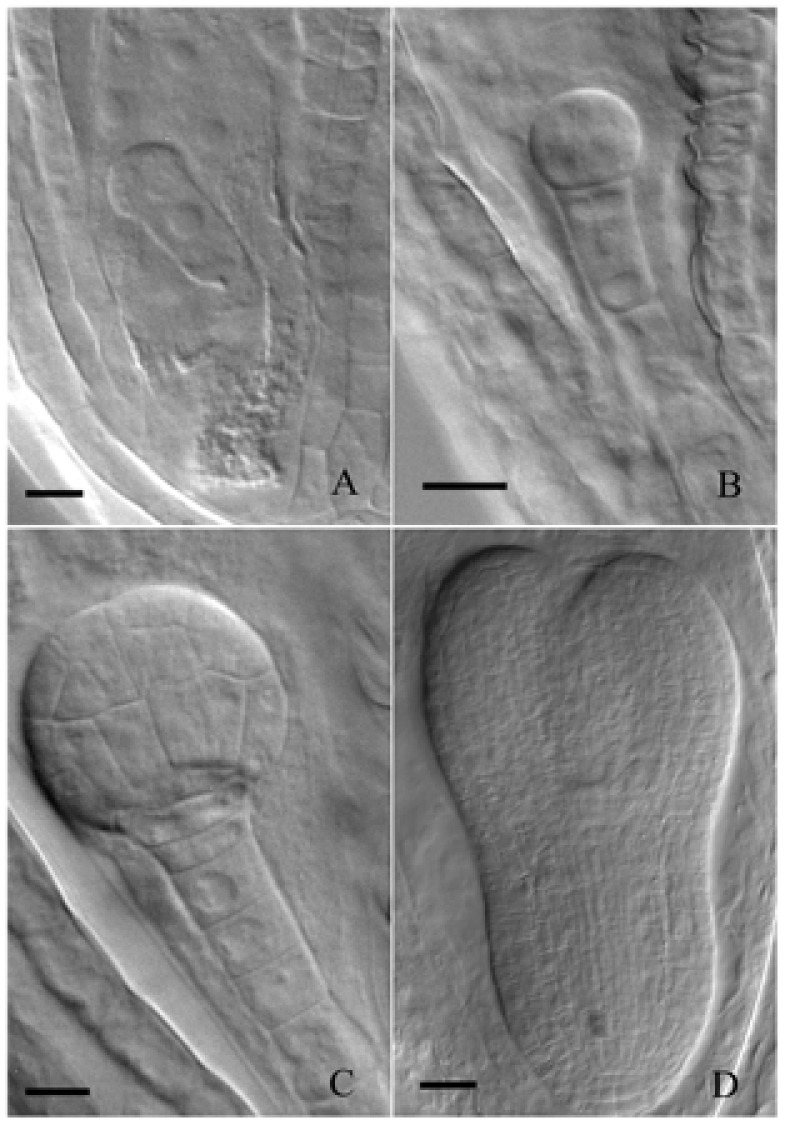
Development of embryos from single-celled zygote to torpedo-shaped embryo. **(A)** Single-celled zygote grown under control conditions. **(B)** Two-celled embryo grown under control conditions. **(C)** Early globular embryo grown under control conditions. **(D)** Torpedo-shaped embryo grown under light deprivation. Bar = 20 μm.

During embryogenesis, plastids in Arabidopsis embryos undergo two opposite processes that is chloroplast formation beginning at the late globular embryo stage and chloroplast dedifferentiation occurring when mature embryos begin to dehydrate (Allorent et al., 2013). In our controlled growth conditions, single-celled zygotes (**Figure 1A**) and two-celled embryos (**Figure 1B**) were observed in most of 1-DAP and 2-DAP ovules, respectively. Early globular embryos were not observed until 3 DAP (**Figure 1C**). Therefore, light deprivation was performed by enwrapping the 3-DAP siliques with tinfoil. Subsequent results showed that torpedo-shaped embryos were observed in both the control and treated 5-DAP ovules (**Figure 1D**). No visible morphological difference was found except that the embryos in tinfoil-enwrapped siliques were not green but quite pale, indicating that light deprivation did not inhibit embryo morphogenesis in Arabidopsis.

**FIGURE 2:**
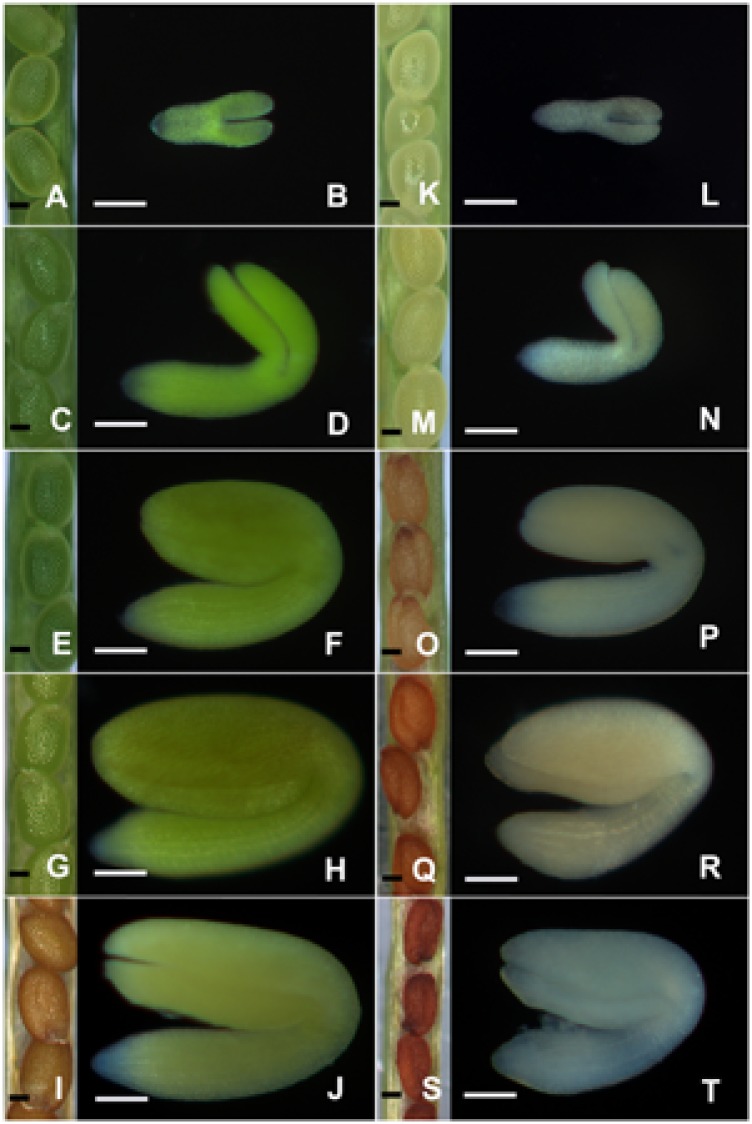
The morphology of ovules/embryos during embryo development. Both the control **(A–J)** and treated **(K–T)** ovules/embryos were analyzed with a Zeiss stereomicroscope when they were grown for 6 **(A,B,K,L)**, 8 **(C,D,M,N)**, 10 **(E,F,O,P)**, 12 **(G,H,Q,R)**, and 14 **(I,J,S,T)** DAP, respectively. Bar = 100 μm.

To further clarify the effects of light deprivation on embryo maturation, comparative studies between the control and treated ovules/embryos at developmental time from 6 to 14 DAP were carried out. The results indicated that control ovules/embryos were changed from light green to green, and finally appeared yellowish/yellow-green when they turned from torpedo to mature embryos (**Figures 2A–J**). On the other hand, although the tinfoil-enwrapped seeds successfully survived torpedo, walking-stick, mature, and even the dormant stages, no green ovules/embryos were observed (**Figures 2K–T**). They changed rapidly from yellowish–white into pale brown or dark-brown with yellowish or white embryos (**Figures 2K–T**), indicating the formation of etiolated embryos in response to light deprivation (Palanisamy and Vivekanandan, 1986a). Besides, in contrast to the control seeds (**Figure 2I**), the seeds obtained from the treated siliques were obviously smaller and shrunken (**Figure 2S**). In fact, the mean fresh and dry weights of the control seeds were about 2.11 ± 0.27 and 1.71 ± 0.18 μg/100 seeds, respectively. Conversely, the respective values for the tinfoil-enwrapped seeds were about 1.09 ± 0.21 and 0.94 ± 0.15 μg/100 seeds, respectively. In other words, light deprivation induced a significant decrease in the fresh and dry weights by approximately 48 and 45%, respectively, as compared with that of the control (*P* < 0.05). In addition, more than 95% of the control seeds reached radicle emergence within 2 days, were developing healthy seedlings. At the same time, the data of the tinfoil-enwrapped seeds only about 29.3% (*n* = 120). Even after 5 days of sowing, the tinfoil-enwrapped seeds still had reduced germination rate (approximately 50%), and developed into obviously weaker seedlings. However, with increasing time of cultivation, the differences between control and treated seedlings disappeared. All these data enable us to conclude preliminary that light deprivation might reduce the accumulation of storage reserves essential for seed maturation and germination.

### Light Deprivation Inhibited Chloroplast Biogenesis during Embryo Development

**FIGURE 3:**
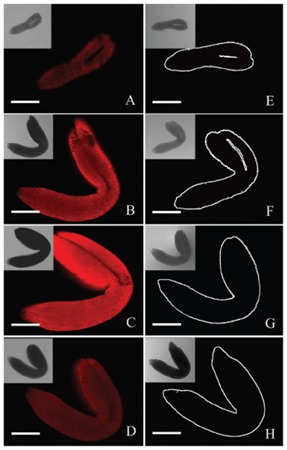
Analysis of chlorophyll fluorescence of control and treated embryos. Both the control **(A–D)** and treated **(E–H)** embryos were analyzed with a laser confocal microscope when they were grown for 6 **(A,E)**, 8 **(B,F)**, 10 **(C,G)**, and 12 DAP **(D,H)**, respectively. Inserts represent corresponding bright field **(B,F)** images. Bar = 100 μm.

Chlorophyll in chloroplasts emits red autofluorescence, which has been widely used as a specific marker to monitor chloroplast development (Feng et al., 2014; Lu et al., 2014). To further elucidate whether light deprivation inhibited chloroplast biogenesis in embryos, a comparative analysis on chlorophyll fluorescence between control and treated embryos was carried out. As shown in **Figure 3**, chlorophyll fluorescence was readily detected in the whole 6-DAP embryos grown under controlled conditions except for radicles (**Figure 3A**). Brighter fluorescence with similar distribution was observed in embryos grown at 8, 10 and 12 DAP (**Figures 3B–D**), with the highest intensity observed in 10-DAP embryos (**Figure 3C**). As a consequence of the sharply decreased signal intensity, only low intensities of chlorophyll fluorescence could be detected in 14-DAP embryos (data not shown). In contrast, though using the same acquisition conditions, hardly any chlorophyll signals could be detected in the tinfoil-enwrapped embryos at all studied developmental stages (**Figures 3E–H**), confirming that light deprivation seemingly prevented the formation of chlorophyll in chloroplasts.

**FIGURE 4:**
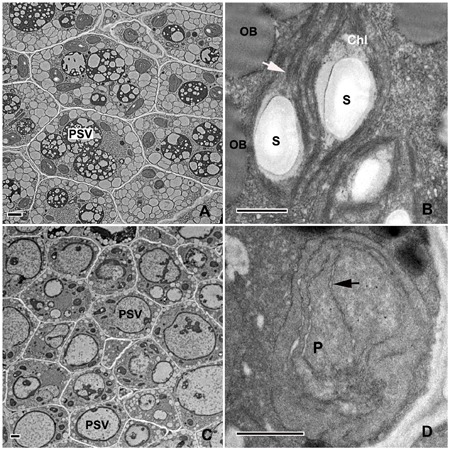
Analysis of the ultrastructure of plastids in 10-DAP embryos. Ultrathin sections of both control **(A,B)** and treated **(C,D)** embryos growing after 10 DAP were analyzed with a Hitachi-7650 transmission electron microscope. The white arrow indicates the internal membrane system (grana and stromal thylakoids in chloroplast. The black arrowhead indicates poorly developed single thylakoid membranes in dark-forced plastids. PSVs, protein storage vacuoles; Chl, chloroplast; P, plastid; S, starch grains; OB, oil bodies. Scale bar: A,B = 1 μm; C,D = 500 nm.

Ultrastructural observation may be the most reliable way to evaluate chloroplast biogenesis. In the present study, the TEM analysis conducted on 10-DAP embryos revealed that cotyledon mesophyll cells of the control embryos contained many plastids with similar sizes and regular shapes (**Figure 4A**). The well-developed grana and stromal thylakoids indicated that these plastids were chloroplasts (**Figure 4B**). On the contrary, although the mesophyll cells of tinfoil-enwrapped embryos also contained large numbers of plastids, they were variable in size and shape, and small starch grains were also sometimes visible (**Figure 4C**). Furthermore, poorly developed single thylakoid membranes were observed in these plastids (**Figure 4D**). Besides, prolamellar bodies (PLBs) were also observed in some of the plastids. All these phenomena enabled us to conclude that they might be etioplasts or proplastids, but not chloroplasts.

### Light Deprivation Reduced the Accumulation of Storage Reserves during Embryo Maturation

**FIGURE 5:**
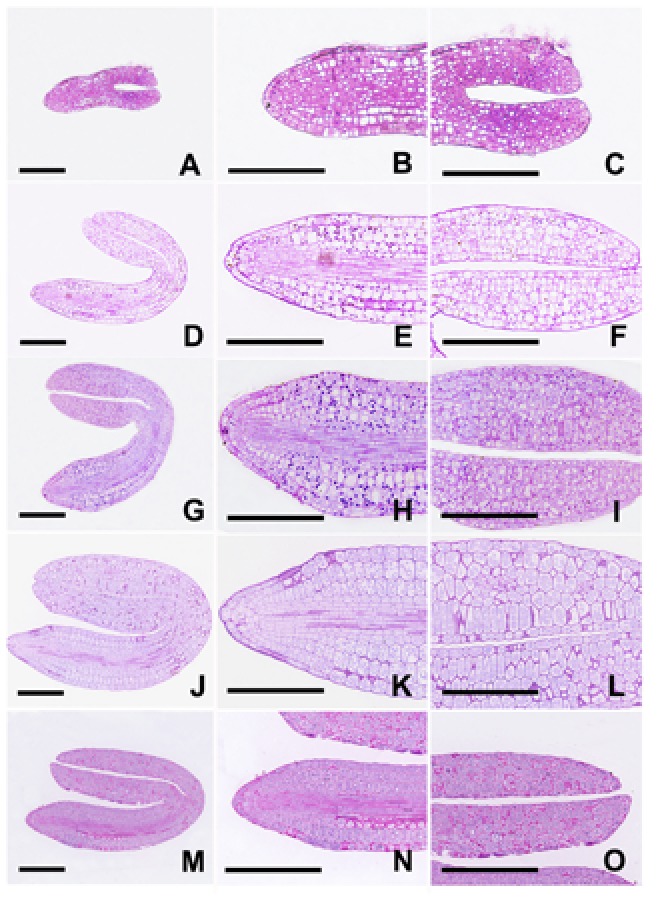
Analysis of starch grains using periodic acid-Schiff reaction. Longitudinal sections of both control **(A–L)** and treated **(M–O)** embryos were prepared and stained with PAS reaction. **(A–C)** Embryos grown under control conditions for 6 DAP. **(D–F)** Embryos grown under control conditions for 8 DAP. **(G–I)** Embryos grown under control conditions for 10 DAP. **(J–L)** Embryos grown under control conditions for 12 DAP. **(M–O)** Embryos grown under light deprivation for 10 DAP. Figures **B–C,** **E–F,** **H–I,** **K–L** and **N–O** represent magnified views of Figures **A, D, G, J** and **M**, respectively. Bar = 100 μm.

Compared with the control, the dark-forced siliques yielded obviously small and shrunken seeds (**Figures 2I,S**), indicating the reduced accumulation of storage reserves in embryos. Since seed’s carbon deposition is believed to be directly affected by the light-induced photosynthesis in chloroplasts (Goffman et al., 2005), histochemical analysis with PAS reaction was carried out in the subsequent studies. As illustrated in **Figure 5**, a few small starch grains were observed in the hypocotyl of 6-DAP control embryos, mainly in the epidermal, cortex and endodermal cells (**Figures 5A,B**). Hardly any starch grains were detected in the 6-DAP cotyledons, even in the mesophyll cells (**Figures 5C**). Subsequently, many starch grains were detected in nearly all cells of the 8-DAP embryos, except for the procambium cells (**Figures 5D–F**). Besides, starch grains in the cotyledons were considerably smaller than those in the hypocotyls. The most and largest starch grains were observed in 10-DAP embryos (**Figures 5G–I**). Starch grains then abruptly disappeared from 12-DAP embryos (**Figures 5J–L**), as well as from 14-DAP embryos (data not shown). In contrast, scarcely any starch grains were found in nearly all tinfoil-enwrapped embryos examined (data not shown), except for the 10-DAP embryos, where a few small starch grains were occasionally observed (**Figures 5M–O**). Light-grown embryos having more starch indicated that photosynthesis in chloroplasts plays important roles in starch synthesis. However, not only chloroplasts are able to produce and store starch.

**FIGURE 6:**
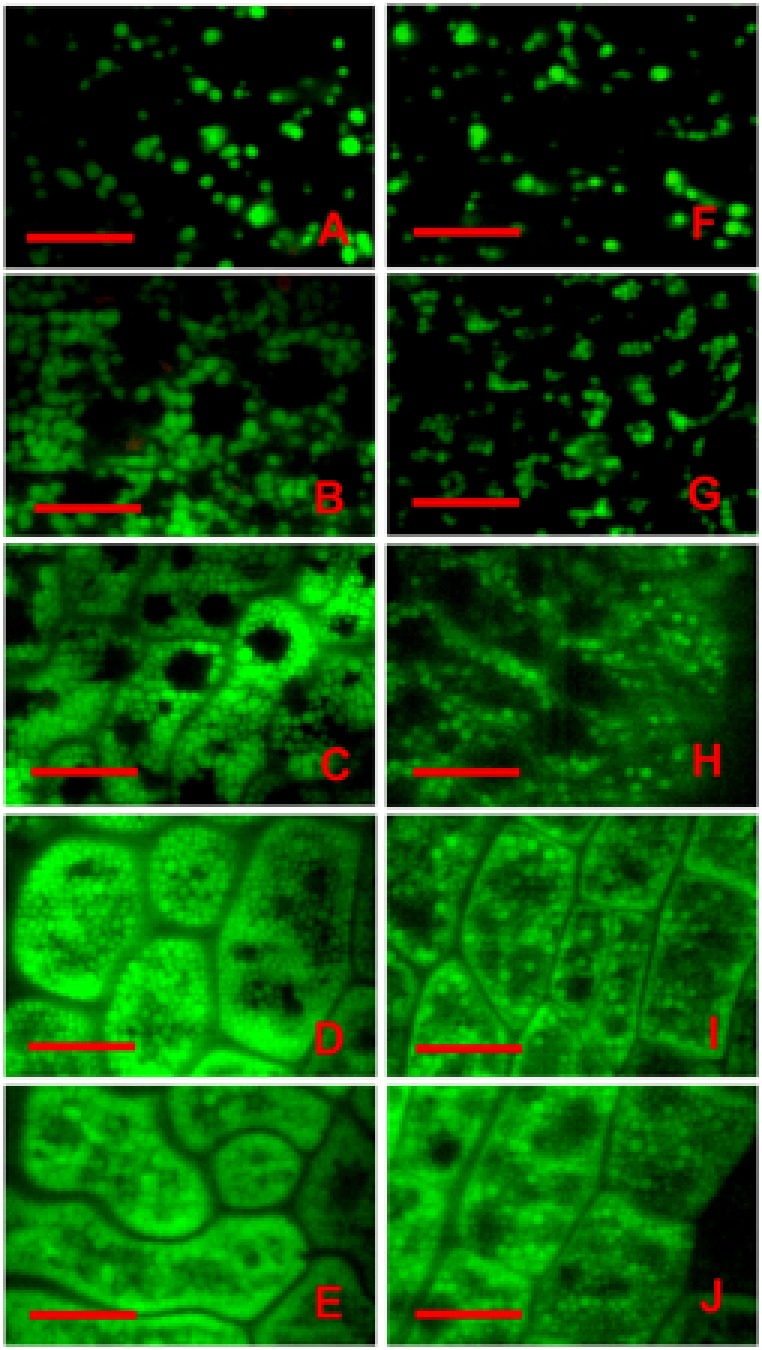
Analysis of oil bodies using Nile red staining. Both the control **(A–E)** and tinfoil-enwrapped **(F–J)** embryos were stained with Nile red, and analyzed with a laser confocal microscope when they were grown for 6 **(A,F)**, 8 **(B,G)**, 10 **(C,H)**, 12 **(D,I)**, and 14 DAP **(E,J)**, respectively. The green fluorescence was emitted from oil bodies stained with Nile red, while the red fluorescence was emitted from chlorophyll in the chloroplast. Bar = 10 μm.

Starch grains represent material that is temporarily stored during Arabidopsis embryo development and is finally being converted into oil (Mansfield and Briarty, 1991). The oil body is a unique oil storage organelle used mainly for storage of triacylglycerol (TAG). Although TAG is synthesized in the endoplasmic reticulum, fatty acids which are essential for the synthesis of TAG, are primarily dependent on the light reaction of photosynthesis in chloroplasts (Asokanthan et al., 1997; Ruuska et al., 2004; Siloto et al., 2006). Therefore we speculated that light deprivation might reduce the accumulation of storage oils during embryo maturation. To investigate this possibility, confocal analysis with Nile red staining of the embryos was performed. Our results showed that spherical oil bodies were already detectible in the control embryos as early as 6 DAP. They were dispersed throughout the cytoplasm and had various sizes (**Figure 6A**). Over time, the oil content increased quickly, as more oil bodies with larger volumes were formed. They tended to be present in the periphery of the cells (**Figures 6B–D**). The maximal amount of oil bodies was observed in the 14-DAP embryos, in which almost the whole cellular space, except for the PSVs and the nucleus, was filled with oil bodies (**Figure 6E**). In contrast, although oil bodies were also detected in the treated 6-DAP embryos, they were obviously fewer and smaller (**Figure 6F**). It is noteworthy that the differences in the oil bodies between the control and treated embryos continuously increased along with embryo development (**Figures 6G–I**). As a result, hardly any cells filled completely with oil bodies were observed in the 14-DAP embryos grown under dark conditions (**Figure 6J**).

**FIGURE 7:**
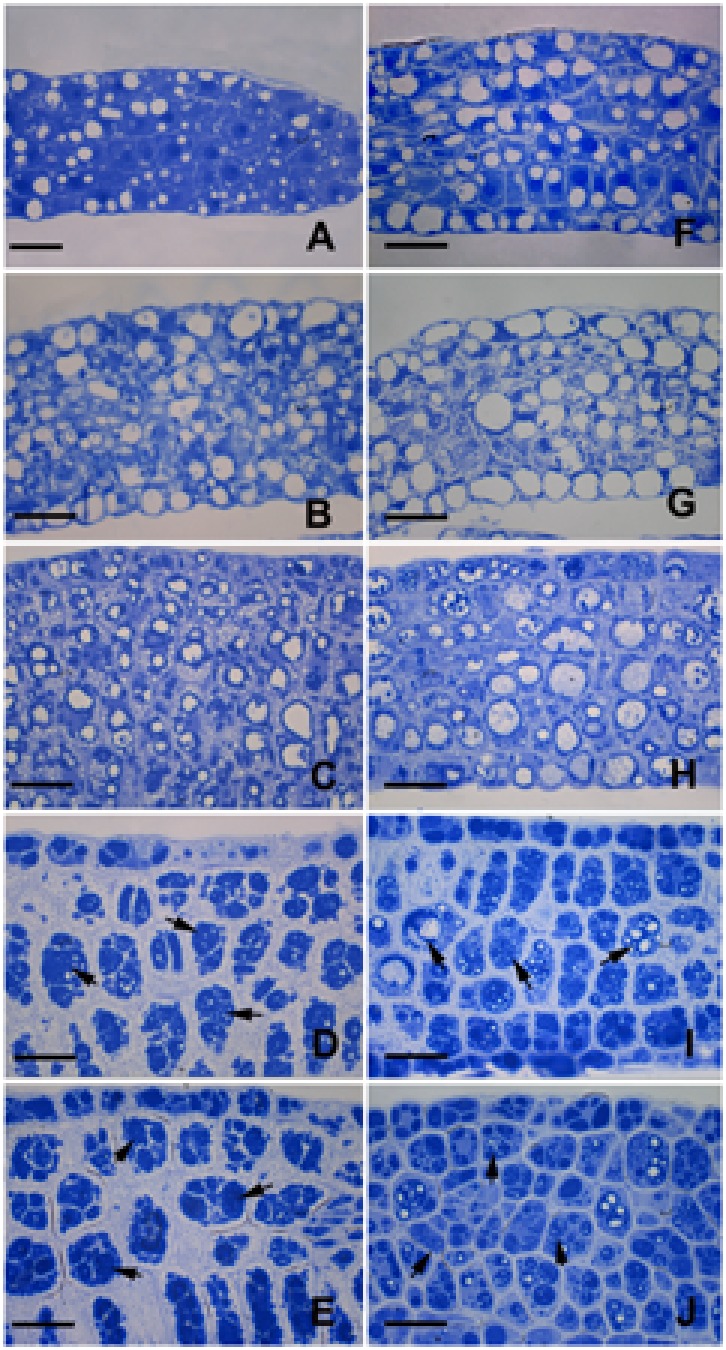
Coomassie brilliant blue staining for total proteins. Semithin sections of both the control **(A–E)** and treated **(F–J)** embryos were obtained and stained with Coomassie brilliant blue. **(A,F)** Embryos grown for 6 DAP. (B,G) Embryos grown for 8 DAP. **(C,H)** Embryos grown for 10 DAP. **(D,I)** Embryos grown for 12 DAP. **(E,J)** Embryos grown for 14 DAP. Arrowheads indicate proteins accumulated in PSVs. Bar = 20 μm.

Proteins are another major reserve storage material in mature Arabidopsis seeds (Mansfield and Briarty, 1991). Thus, storage protein accumulation was also evaluated using Coomassie brilliant blue staining of the slides. In embryos grown under control conditions for 6 DAP, small amounts of storage proteins were observed in the small protein storage vacuoles (PSVs) (**Figure 7A**). Subsequently, larger PSVs with some storage proteins were frequently detected in 8-DAP embryos (**Figure 7B**). As the protein content increased, a continuous peripheral layer of protein was formed at the inner surface of PSVs in the 10-DAP embryo cells (**Figure 7C**). Finally, PSVs were almost completely filled by storage proteins (**Figures 7D,E**), except for some small irregular spaces (Mansfield and Briarty, 1991). Usually, the vast majority of cytoplasmic proteins in the mature seeds were transported into PSVs located at the center of cells, whereas oil bodies, which were hard to be stained by Coomassie brilliant blue, were present in the periphery of the cells and between PSVs (**Figures 7D,E**).

On the other hand, although the formation of PSVs was indeed observed in the embryos growing under dark conditions (**Figure 7F**), significant differences between the control and treated embryos were also established. Especially, the original accumulation of proteins was not mainly peripheral; instead, proteins were often dispersed throughout the PSVs (**Figures 7G,H**). Besides, most of the PSVs in the treated embryos were less dense than those in the control (**Figures 7H–J**). At the same time the cytoplasm of the treated embryos was still markedly stained by the Coomassie brilliant blue, which was hardly observed in the control embryos.

**FIGURE 8:**
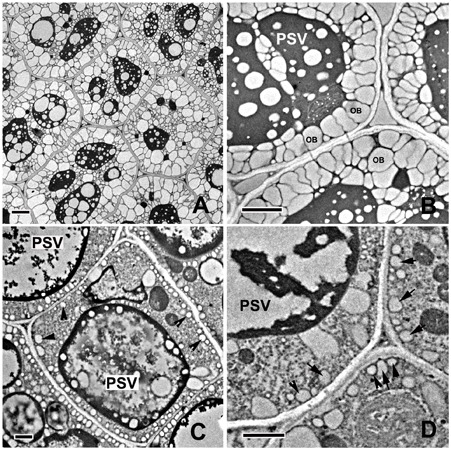
Analysis of the ultrastructure of the storage reserves in mature embryos. Ultrathin sections of both control **(A,B)** and treated **(C,D)** embryos growing for 14 DAP were analyzed with a Hitachi-7650 transmission electron microscope. Arrows show the oil bodies in dark-forced embryos. PSVs, protein storage vacuoles; OB, oil bodies. Bar: A,B = 2 μm; C,D = 1 μm.

To further confirm the results of the observations under the light microscope, TEM observation on 14-DAP embryos was also performed. As shown in **Figures 8A,B**, the PSVs in embryos growing under normal conditions were almost completely filled by storage proteins except for small electron-transparent ‘bubbles’ that were presumably areas from which globoids had been lost during tissue processing (Mansfield and Briarty, 1991). Large oil bodies were observed mainly at the periphery of the cells (**Figures 8A,B**). The electron-dense deposits in the PSVs of the treated embryos were substantially less than those of the control ones (**Figures 8C,D**). Furthermore, only a few small oil bodies were observed at the periphery of the cells of the treated embryos. All these data confirmed that light deprivation did reduce the accumulation of storage reserves during embryo maturation.

## Discussion

Light is an essential environmental factor for the formation of chloroplasts from other types of plastids such as proplastids, as well as leucoplasts, etioplasts and chromoplasts. Under continuous dark conditions, proplastids develop into etioplasts, instead of chloroplasts, as a consequence of inhibited chlorophyll biosynthesis and chloroplast biogenesis (Solymosi and Schoefs, 2010). Etioplasts usually contain carotenoids and low amounts of protochlorophyllide (Pchlide), with an inner membrane system sometime organized into highly regular PLBs. During the past decades, dark treatment has been widely used in research on the roles of chloroplasts in plant vegetative organs, especially in the leaves (Kutschera and Briggs, 2013). On the other hand, few studies have been carried out on the effects of darkness-induced inhibition of chloroplast development during embryogenesis, except that masking of fruits was reported to result in the formation of etiolated embryos in *Dolichos lablab* L. (Palanisamy and Vivekanandan, 1986a). In the present study, light deprivation was performed by enwrapping the siliques with tinfoil. Anatomical observation revealed that although the tinfoil-enwrapped embryos successfully survived and underwent subsequent development (**Figures 1, 2**), neither green ovules/embryos nor chlorophyll autofluorescence was observed in the dark-forced siliques (**Figures 2, 3**). These results were similar to a previous report that dark treatment induced etiolation in embryos of *D. lablab* L. (Palanisamy and Vivekanandan, 1986b). Furthermore, TEM analysis revealed that although the mesophyll cells of tinfoil-enwrapped embryos also contained large numbers of plastids, the poorly developed single thylakoid membranes indicated that they might be etioplasts or proplastids, but not chloroplasts (**Figures 4**). The pigment composition and chlorophyll biosynthesis of these dark-forced embryos may need further investigation.

Since chloroplasts are present in nearly all green plant parts except the roots, plastid-related mutations usually disturbed the functions of chloroplasts throughout the plant (Inaba and Ito-Inaba, 2010). Kim et al. (2013) reported that mutation in chloroplast-related proteins induced arrest of the embryo, but it was rescued by supplementation with sucrose or glucose, indicating that an embryo arrest might result from insufficient amounts of assimilates provided by leaves and other green parts of the mother plant (Mansfield and Briarty, 1991). In the present study, the light deprivation-induced inhibition of chloroplast biogenesis was limited to a part of the siliques. The chloroplasts in the other parts of the plant were unaffected. Therefore, it was reasonable for us to suggest that our specific growth conditions had an advantage to illustrate the roles of the chloroplasts present in embryos, rather than in the whole plant, on embryogenesis.

The embryos of many plants contain chloroplasts, and are referred to as chloroembryos (Yakovlev and Zhukova, 1980; Puthur et al., 2013). The spatial differentiation and distribution of chloroplasts in embryos is believed to be of crucial importance during embryogenesis, which has been indeed supported by many previous reports (Hsu et al., 2008; Wang et al., 2010; Trosch and Jarvis, 2011; Yin et al., 2012; Feng et al., 2014; Lu et al., 2014; Chen et al., 2015). The disruption of the functions of chloroplasts has been frequently associated with embryo lethality.

On the other hand, not all mutants defective in chloroplast functions inevitably resulted in embryo lethality. For example, the Arabidopsis albino mutant deficient in chlorophyll was able to produce germinable seeds (Sundberg et al., 1997). Mutation in monogalactosyldiacylglycerol (MGDG) synthase 1 has been found to result in seeds producing small albinos with disrupted photosynthetic membranes (Kobayashi et al., 2007). Besides, a mutation in EMB1211 influenced chloroplast biogenesis, leading to the lethality of young seedlings (Liang et al., 2010). Similarly, the disruption of PPR8522, a chloroplast-targeted protein, caused an albino, seedling-lethal phenotype in maize (Sosso et al., 2012). It was therefore suggested that embryo development would be arrested when the biosynthetic functions or the expression of the chloroplast genome were inhibited, but embryo development would proceed when the functions of photosynthesis were disrupted (Bryant et al., 2011). In the present study, although no chloroplasts were observed in tinfoil-enwrapped ovules, the embryos successfully survived and continued their development (**Figures 1–4**). Our results are consistent with a previous report revealing that an *in vitro* culture of Arabidopsis embryos at the 4- and 16-cell stages, grown under dark conditions, developed fully into mature albino embryos (Sauer and Friml, 2004), indicating that chloroplast functions may not be essential for seed development.

More than 30% of the genes required for proper embryo formation were believed to encode plastid-targeted proteins (Hsu et al., 2010), reflecting vital roles of plastid proteins in embryogenesis. Nevertheless, many of these genes are not directly related to chloroplasts. In fact, some of them were found to be necessary for the development of preglobular embryos and/or their conversion to globular embryos, when the embryos still did not contain chloroplasts (Hsu et al., 2008, 2010; Inaba and Ito-Inaba, 2010). Given that the interconversions among proplastids, chloroplasts, etioplasts and leucoplasts are reversible, the chloroplast-localized proteins might be probably required not just for chloroplast function but also likely for the functioning of other plastids. Thus, we may further speculate that plastids rather than chloroplasts were essential for embryo development. Besides, light deprivation indeed inhibited the formation of chloroplasts. In this sense, our methods might also have the advantage of illustrating specifically the roles of chloroplasts on embryogenesis rather than those of all kinds of plastids.

In this study, although fully developed seeds were obtained from tinfoil-enwrapped siliques, they were obviously small and shrunken, and with reduced germination capacity (**Figure 2**). Our results are partially reminiscent of those of a previous report evidencing that biomass accumulation in seeds in the dark was less than one third of that in light (Goffman et al., 2005), indicating that disturbance in chloroplast functions may affect reserve deposition essential for embryo maturation and seed germination.

Lipids are important reserves generally stored as TAGs in oil bodies (Murphy, 1990; Siloto et al., 2006). The use of fatty acids for the synthesis of TAG is energetically expensive, requiring a large amount of ATP, NADPH, and NADH (Goffman et al., 2005; He and Wu, 2009; Nakajima et al., 2012). It was proposed that the photosynthetic electron transport in embryos can provide NADPH and ATP for fatty acid synthesis under ambient light conditions (Ruuska et al., 2004). Furthermore, a positive correlation was found between enhanced illumination and the marked stimulation of lipid accumulation, whereas the inhibition of photosynthesis strongly reduced oil synthesis (Ruuska et al., 2004; Goffman et al., 2005). In the present study, both Nile red staining and TEM observation showed that light deprivation strongly inhibited the accumulation of lipids in embryos (**Figures 6, 8**). Our data are consistent with, and confirm the findings of previous reports that the light reaction of photosynthesis in chloroplasts is of critical significance for lipid accumulation in embryos.

The conversion of carbon supplies into lipids induces a substantial loss of carbon as CO_2_. In fact, 33% of the carbon supplied to fatty acid synthesis is believed to be potentially released as CO_2_ (Goffman et al., 2005). This markedly reduces the carbon storage efficiency if no mechanisms of CO_2_ refixation are present in embryos. Previous reports have indicated that green seeds are photosynthetically active and are able to fix carbon dioxide. Goffman et al. (2005) reported that light significantly increased the embryo growth and carbon conversion efficiency while reducing the loss of carbon as CO_2_. It was even proposed that seed photosynthesis functions in simply fixing CO_2_ in developing seeds (Albrecht et al., 2008). On the other hand, Browse and Slack (1985) reported that photosynthesis did not significantly contribute to the carbon economy of the developing seed. Eastmond et al. (1996) suggested that the photosynthesis in embryo plastids made no net contribution to carbon economy *in vivo*. Similarly, Asokanthan et al. (1997) concluded that the primary role of chloroplasts in embryos was not to photoassimilate CO_2_. In this investigation, an increase in the amount and size of starch grains was observed in the stages from torpedo to mature embryos, but starch abruptly disappeared thereafter (**Figure 5**). This indicates that the accumulation of starch grains was tightly associated with the developmental status of chloroplasts in embryos (**Figure 3**). Furthermore, hardly any starch grains were observed in nearly all tinfoil-enwrapped embryos examined (**Figure 5**). This provides a direct evidence that chloroplasts significantly increased carbon conversion efficiency during embryo development.

Protein is another main storage substance in the seeds (Mansfield and Briarty, 1991; Herman and Larkins, 1999). Increasing light irradiance was shown to promote the accumulation of proteins in embryos (Goffman et al., 2005). In the present study, fewer proteins were observed in PSVs under dark conditions. By contrast, the cytoplasm of mature embryos growing under dark condition could easily be stained with Coomassie brilliant blue unlike those of the control embryos (**Figure 7**), implying that light deprivation affected the deposition of proteins in seeds. Yet, we still cannot explain the precise mechanism leading to this phenomenon. The most probable explanation is that the inhibition of chloroplast biogenesis influences the synthesis of amino acids essential for protein synthesis (Goffman et al., 2005). Alternatively, delayed protein transportation in response to the absence of the light reaction of photosynthesis may be another possibility that cannot be excluded.

In short, our data indicate that light deprivation could effectively inhibit the formation of chloroplasts in Arabidopsis embryos, and that the inhibition of chloroplast development did not arrest embryo morphogenesis, but seriously reduced the accumulation of storage reserves during embryo maturation in Arabidopsis.

## Author Contributions

XS designed the research. HL, XW, KR, and KL performed research. XW, MW, and WW analyzed data. HL, XW, KR, and XS wrote the paper.

## Conflict of Interest Statement

The authors declare that the research was conducted in the absence of any commercial or financial relationships that could be construed as a potential conflict of interest.
