# Corrigendum: Light Deprivation-Induced Inhibition of Chloroplast Biogenesis Does Not Arrest Embryo Morphogenesis but Strongly Reduces the Accumulation of Storage Reserves during Embryo Maturation in Arabidopsis

**DOI:** 10.3389/fpls.2017.02250

**Published:** 2018-02-06

**Authors:** Frontiers Editorial Office

**Affiliations:** Frontiers Media SA, Switzerland

**Keywords:** *Arabidopsis thaliana*, chloroplast, embryogenesis, storage reserves, oil body, protein storage body

In the published article there was an inappropriate term in the Results section, Light Deprivation Did Not Inhibit Embryo Morphogenesis but Affected Embryo Maturation sub-section, Paragraph 2. The paragraph should read:

To further clarify the effects of light deprivation on embryo maturation, comparative studies between the control and treated ovules/embryos at developmental time from 6 to 14 DAP were carried out. The results indicated that control ovules/embryos were changed from light green to green, and finally appeared yellowish/yellow-green when they turned from torpedo to mature embryos (Figures 2A–J). On the other hand, although the tinfoil-enwrapped seeds successfully survived torpedo, walking-stick, mature, and even the dormant stages, no green ovules/embryos were observed (Figures 2K–T). They changed rapidly from yellowish–white into pale brown or dark-brown with yellowish or white embryos (Figures 2K–T), indicating the formation of etiolated embryos in response to light deprivation (Palanisamy and Vivekanandan, 1986a). Besides, in contrast to the control seeds (Figure 2I), the seeds obtained from the treated siliques were obviously smaller and shrunken (Figure 2S). In fact, the mean fresh and dry weights of the control seeds were about 2.11 ± 0.27 and 1.71 ± 0.18 μg/100 seeds, respectively. Conversely, the respective values for the tinfoil-enwrapped seeds were about 1.09 ± 0.21 and 0.94 ± 0.15 μg/100 seeds, respectively. In other words, light deprivation induced a significant decrease in the fresh and dry weights by approximately 48 and 45%, respectively, as compared with that of the control (*P* < 0.05). In addition, more than 95% of the control seeds reached radicle emergence within 2 days, were developing healthy seedlings. At the same time, the data of the tinfoil-enwrapped seeds only about 29.3% (*n* = 120). Even after 5 days of sowing, the tinfoil-enwrapped seeds still had reduced germination rate (approximately 50%), and developed into obviously weaker seedlings. However, with increasing time of cultivation, the differences between control and treated seedlings disappeared. All these data enable us to conclude preliminary that light deprivation might reduce the accumulation of storage reserves essential for seed maturation and germination.

The original article has been updated.

## Conflict of interest statement

The authors declare that the research was conducted in the absence of any commercial or financial relationships that could be construed as a potential conflict of interest.
